# Positive effects of COVID-19 on food preparation and expenditure habits: a comparative study across three countries

**DOI:** 10.1017/S1368980022001720

**Published:** 2022-11

**Authors:** Asli Emine Özen, Asker Kartarı, Antonia Correia, Jun Wen, Metin Kozak

**Affiliations:** 1 Department of Nutrition and Dietetics, Final International University, Girne, North Cyprus, Turkey; 2 School of Communication, Kadir Has University, Istanbul 34083, Turkey; 3 Faculty of Economics, University of Algarve, Algarve, Portugal; 4 School of Business and Law, Edit Cowan University, Perth, Australia

**Keywords:** Food preparation, Food expenditure, Cross-cultural research, China, Portugal, Turkey

## Abstract

**Objective:**

This study seeks to empirically investigate how the changing eating habits affect health habits within three countries with entirely different cultures and diets to understand to what extent the pandemic may be responsible for these changes.

**Design:**

Specifically, a questionnaire was conducted in China, Portugal and Turkey in early 2021. A series of statistical analyses were performed to identify how changes in individuals’ eating habits have influenced their diets, considering the pandemic context and the varying cultural contexts where this research was performed.

**Setting:**

A structured questionnaire form was developed and uploaded to an online platform with unique links for automatic distribution to respondents in each country. Data for the main survey were gathered between 3 January and 1 February 2021.

**Participants:**

Using snowball sampling, the authors leveraged their social networks by asking friends and colleagues to distribute the survey to potentially interested individuals. This distribution was stratified accordingly to the distribution of the population. The authors ultimately collected 319 useable surveys from China, 351 from Portugal and 449 from Turkey.

**Results:**

The pandemic inspired healthier food habits, mostly because people have additional time to cook, shop differently for food and spend more money on groceries.

**Conclusions:**

The study suggests that aside from cultural values and dietary habits, the available time and the fear of the pandemic most explained the new eating habits. Several implications are provided for researchers and overall society in these three countries.

Technological advances and globalisation have led the world market to become more open during the 21st century. The emergence of COVID-19 unequivocally altered individuals’ daily lives in affected countries on a micro-level (e.g. household economies and attitudes) and macro-level (e.g. national economies and cultural values). The effects of COVID-19 on daily life were researched^(1,2)^; nevertheless, little research has been done using a cross-country approach, and in particular, in countries with entirely different cultures, to depict the extent to which healthy eating habits are changing because of the pandemic, also based on countries’ cultures and habits^(3)^.

The eating cultures of these countries are far from similar. Chinese cuisine comprises rice or noodles, vegetables and meat, and soup as main dishes^(4)^. A Turkish meal includes soup, vegetables, meat or legumes. Part of the meal is also rice, bulgur, pasta, salads and yoghurt with cucumbers and garlic^(5)^. On the other hand, Portuguese meals start with appetisers, a main dish of meat, fish or vegetables, and dessert^(6)^. Slight differences are perceived in the menu composition. For instance, in Portugal, the Mediterranean tradition of having meat or fish as the main dish is complemented with an extra portion of carbohydrates – rice and potatoes are usually served simultaneously^(7)^. In China, the cooking techniques and flavours distinguish the Chinese eating habits^(4)^. Turkish eating habits differ in the consumption of fats and oils^(8)^ and the cooking techniques such as stewing, frying, grilling, roasting and baking.

This study assumes a micro-perspective to investigate how the change in eating habits in three countries with quite different cultural habits and diets impacts citizens’ health habits. This cross-cultural research allows a dismantling of the impact of culture from that of the pandemic on residents’ eating habits in different cultural settings. To this end, an ordered probit model was estimated for each country to understand how changes in food habits have influenced individuals’ healthy eating. Recommendations are provided for researchers and the general public in these countries. The main contributions of this research are theoretical as it proposes a model that could be tested in different settings and scientific as this is the first research to dismantle cultural and pandemic effects under a cross-cultural setting empirically, and the present results recommendations to the society.

## Literature review

Most people have experienced dramatic life changes during COVID-19. Restrictions have forced people to adapt to a ‘new normal’. Being confined at home during the pandemic may have led some people to cope with stress and anxiety by devoting more time to food preparation, cooking and preservation. Having more time but preferring to shop for food less frequently may have also driven households to preserve food for future consumption. The following sections outline changes in individuals’ food purchases, preservation, preparation, cooking and expenditure.

## Possible changes in shopping habits

Many scholars have observed that lockdown influenced food purchases. Grocery store visits decreased during the lockdown in the Netherlands^(9)^ and the USA^(10)^. Many studies have shown that online grocery shopping has become a primary means of food acquisition^(9)^. The food industry has also faced radical changes due to partial lockdowns, restrictions on in-store capacity and reduced operating hours. Some restaurants remained closed for months and needed to implement new approaches to service delivery, such as online ordering^(11)^ and the development of mobile applications for such services^(12)^. People have made use of credit cards more intensively for online shopping, rather than going out to spend directly in restaurants and groceries^(13)^.

In Turkey, for instance, companies improved their existing applications to accept online orders from households (e.g. *GetirBüyük*). An online food delivery company also expanded its market potential (e.g. *Yemeksepeti*). To increase the delivery service, the supermarkets used taxis paid for by the municipalities in Portugal. Online purchases accelerated in China due to the COVID-19 pandemic. Consumers’ purchase preferences (e.g. cosmetics) also favoured contactless, rather than in-person, transactions^(14)^. Wen *et al.*^(2)^ found that Chinese diners’ likelihood of having face-to-face dining declined during the pandemic. Elsewhere, countries such as India pioneered several online food delivery applications such as *Swiggy* and *Zomato*. These players are likely to improve their market share and threaten regular restaurants, with some establishments even deciding to cease operations. While there has been no indication of how long such practices may continue or their dominance in the market, specific population segments (e.g. the elderly and office workers) have benefited greatly.

## Possible changes in food preservation

When the WHO declared COVID-19 a pandemic in March 2020, disruptions in the food chain resulted in limited access to fresh food^(15)^. This unforeseen outcome led to food insecurity; food was available but not necessarily accessible. The FAO defines food security as having consistent access to the food necessary for a healthy life. Food security is based on four pillars: ensuring a safe and nutritionally adequate food supply, stability in the food supply, food availability, and social and economic access to sufficient food^(16)^. Food preservation allows foods to retain their quality and be stored for longer periods and even throughout the year. Therefore, preserved foods can offer a practical solution to sustain a stable and adequate supply of and access to food. Today’s food industry uses different preservation techniques to produce nutritious and safe foods with a long shelf life. For this reason, traditional food preservation skills have been in decline in recent decades^(17)^. This trend may explain why consumers bought food with a long shelf life early in the pandemic^(15)^. Individuals can also store preserved food longer, resulting in less frequent trips to the grocery store.

In Portugal, the traditional ways of food preservation are no longer used, and freezing methods replaced dried fish, salt conservation and bulk conservation. This preservation increased substantially when the food shortage panic started with the first lockdown. As one of the oldest methods for food preservation, drying is still practised by the rural population in Turkey. Many fruits and vegetables are seasonally abundant and cheaper, so rural people prefer to dry these fruits and vegetables. They consume such dried food during winter and sell them to earn money. Moreover, pickling or jam preparation is still practised by many urban populations. Panic buying at the beginning of the pandemic caused a food shortage. Some people then began to buy fresh foods and preserve them in the case of further shortages. The most straightforward and economical food preservation method is home freezing, which does not require specialised equipment (e.g. a canning machine or dehydrator) and requires limited preparation. Compared with other preservation methods, frozen foods also maintain their quality and nutritional value^(18)^.

## Possible changes in food preparation

In the 20th century, alongside lifestyle changes and the entry of many women into the workforce, home cooking habits changed. Several studies have indicated that time spent by individuals on food preparation and cooking has declined in recent decades^(19,20)^. With increased availability and accessibility of ultra-processed foods (UPF), the time required to prepare and cook meals have decreased. Moreover, ultra-processed foods have enabled people to prepare meals with less skill. Meals eaten outside the home have increased simultaneously^(21)^. A decline in home cooking has contributed to nutritional concerns due to the adverse effects of consuming ultra-processed or takeaway foods. Several studies have shown that the consumption of ultra-processed foods is associated with an increased risk of diet-related diseases^(22,23)^. Likewise, takeaway foods often correspond to a higher intake of calories, fat and Na but a lower intake of fruits, vegetables and wholegrains^(21)^ and a poorer diet quality^(24)^.

Studies showed that the consumption of UPF increased in Portugal^(22)^ and China^(25)^. While UPF contributes 23·8 % of total energy intake among Portuguese^(26)^, in China, UPF provides 18 % of total energy^(27)^. Flavoured yoghurt, cold meat and soft drinks are the main preferred UPF among Portuguese^(26)^; instant pork-mince steam bun/dumpling, instant noodles, cookies, cakes, sausages and packed snacks are the most commonly consumed UPF among Chinese^(25)^ and confectionery, sweet biscuits, soft drinks and cold meats are the main UPF consumed by Turks^(28)^.

Many studies have reported a rise in home cooking during the pandemic^(29)^ as government lockdowns forced people to spend more time at home. In addition, restaurants were shut down or offered only takeaway during these periods. Research has indicated that home cooking is associated with a healthier diet^(19)^, including greater consumption of fruit and vegetables^(30)^. In addition to the nutritional benefits of home cooking, a systematic review summarised other positive outcomes, for example, the development of personal relationships and stronger gender or cultural identities^(31)^.

## Possible changes in cooking style

Perhaps regardless of country-based differences, more time at home has given people a chance to bake, try new recipes and practise different cooking styles. For instance, time-consuming tasks such as baking increased during lockdown^(29)^. Therefore, the pandemic has resulted in a transition to healthy cooking and food preparation: cooking more often, cooking with fresh ingredients and eating takeaway less frequently^(32)^. Parents reported cooking more meals from scratch during the pandemic^(33)^.

## Possible changes in food expenditure

A large body of empirical evidence and secondary sources have addressed the pandemic’s adverse effects on individuals’ well-being and comfort, cultural values, and economic conditions worldwide^(2)^. Despite limited insight into how the pandemic has affected individuals’ spending on at-home food consumption, COVID-19 appears to have adversely affected families with food allergies due to an increase in food prices and a lack of food availability; monthly spending among this group has increased by 23 % during the pandemic^(34)^.

The possible impacts of the pandemic on food-related expenditure span multiple categories. First, given the severe effects of COVID-19 on national economies, millions of citizens lost their jobs or earned less income compared to previous years. This trend may have directly resulted in lower food consumption expenses. Second, empirical evidence suggests that people are motivated to dine out for social interaction, leisure, pleasure and work activities^(35)^, although such behaviour varies across countries. However, long-term lockdowns forced households to stay home and prevented them from dining out with family or friends. So, individuals may have spent less on eating out but spent more on food for at-home consumption.

Third, as a direct consequence of more people living in the same household, total spending on at-home food consumption is likely to have increased. From a macro-economic perspective, some countries have an inadequate food supply to meet the increasing demand. This problem may have led food prices to increase substantially, leading to unpredictable jumps in inflation rates both nationally and internationally. Furthermore, income-related uncertainty may have compelled individuals to save more money, which may have been spent on food (e.g. to enjoyably meet a basic human need). Finally, changes in food expenditure could have been influenced by age and personal preferences (e.g. being a vegetarian or meat-eater)^(36)^.

## Methodology

Based on the findings of earlier studies regarding the pandemic’s effects on consumer behaviour^(34)^, the survey distributed in this study was intended to investigate how COVID-19 may have inspired changes in individuals’ food shopping, preservation, and preparation, cooking, and expenditure. The questionnaire has been described in detail elsewhere^(37)^.

**Fig. 1:**
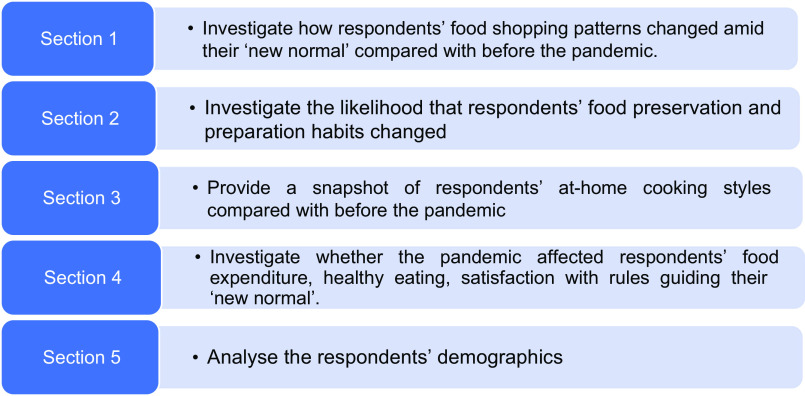
Steps in survey design

As indicated in Fig. 1, the survey consisted of five parts. The first section focused on how respondents’ food shopping patterns changed amid their ‘new normal’ compared with before the pandemic. The second section investigated the likelihood that respondents’ food preservation and preparation habits changed. The following section provided a snapshot of respondents’ at-home cooking styles compared with before the pandemic. The fourth section pertained to whether the pandemic affected respondents’ food expenditure, healthy eating, satisfaction with rules guiding their ‘new normal’ and physical activity. These sections were developed with items measured with a five-point agreement scale, comparing the current situation with the previous one. The last section solicited respondents’ demographics.

This questionnaire was uploaded to an online platform with unique links for automatic distribution to respondents. Data for the main survey were gathered between 3 January and 1 February 2021. Each author was responsible for approaching potential respondents in their respective countries by sending separate emails to each respondent. Using snowball sampling, the authors leveraged their social networks by asking friends and colleagues to distribute the survey to potentially interested individuals. This distribution was stratified accordingly to the distribution of the population. The first respondents were part of the social networks of the researchers, but this relation was lost as more people were involved in sharing the questionnaire. Only one person per household was asked to participate in the survey. Once data collection was discontinued, all questionnaires were checked for missing variables. Surveys containing more than five unanswered items, and those from eleven respondents who had responded incorrectly to an attention check question, were discarded from the analysis. The remaining items were merged into a single table to run statistical analysis and explore possible differences within or between countries. The authors ultimately collected 319 useable surveys from China, 351 from Portugal and 449 from Turkey.

Empirical data were analysed using factor analysis and non-parametric tests to examine differences between the three countries. An ordered probit model was constructed to discern the effects of changes in food habits on individuals’ healthy eating. The analysis consisted of five steps. Exploratory factor analysis (EFA) was conducted to identify dimensions and constructs from the data, as no prior studies had tested these features together. Factor extraction involved maximum likelihood estimation with varimax rotation. The analysis applied a latent root criterion of 1·0 for factor insertion; 0·5 was the cut-off criterion for factor extraction. The second step involved rescaling the constructs extracted using a five-point scale. The means of all components within each construct were used. Once the constructs were derived from EFA and converted into a three-point scale, regression analysis was used. As the variables were ordinal, an ordered probit model was deemed suitable. The model cut-off for the first category was ‘not important at all’. Stata 13 was used to estimate the model through a maximum likelihood function. The second analysis step involved estimating a general model from which individual country models were derived before validating the general model.

The third step entailed independent-sample Kruskal–Wallis tests of extracted components to determine whether the distribution of all samples was the same. The fourth step comprised pairwise comparisons of groups of two countries to test whether each sample distribution coincided. The fifth step involved estimating an ordered probit model, adjusted for the entire sample and estimated for each country, to determine how the changes in food habits had affected individuals’ healthy food, dismantling the cultural habits each country represents.

## Results

Data were gathered in China, Portugal and Turkey with roughly the same distribution. The authors initially intended to approach 314 respondents in each country, given the assumption of a binomial distribution with a maximum dispersion; that is, at least half of the population was expected to alter their food habits during lockdown with a CI of 95 % and a sampling error of 2·5 %. Among the 319 valid questionnaires from China, 351 from Portugal and 449 from Turkey, the sampling error was lower than anticipated and thus ensured better generalisability of the results.

The sample profile in each country can be summarised as follows. On average, respondents were 43·0 years old in Portugal, 42·5 years old in Turkey and 30·0 years old in China. Most respondents in China were between 18 and 34 years old (76·4 %). Many of those in Portugal (75·8 %) and two-thirds in Turkey (65·7 %) were between 35 and 64 years old. Women were nearly identically represented in each country’s sample: 66·0 % (China), 67·0 % (Portugal) and 65·0 % (Turkey). The sample distribution across ages is very similar to the distribution of the population by age in those countries. As such, it could be assumed that the sample is generalisable. Most respondents held a full-time job: 57·0 % (China), 82·0 % (Portugal) and 61·0 % (Turkey). Many respondents in China were students (25·0 %), and a fair proportion in Turkey was retired (17·0 %). Nearly half of the respondents lived with two or three other people. One-third of Chinese respondents lived with four to six other people (33·8 %), whereas one-quarter lived with one person in Portugal and Turkey. Regarding the risk of spreading COVID-19, Turkey ranked first in terms of family members who had tested positive (12·5 %), followed by Portugal (7 %) and China (0·5 %).

### Part I – comparison across China, Portugal and Turkey

As noted above, this study was composed of two main parts. The first part involved an overview of how the pandemic has led to potential changes in shopping habits, food preservation, food preparation, cooking styles, food expenditure, length of stay at home and demographic characteristics among individuals in China, Portugal and Turkey. Comparative results in each category are summarised below.

#### Changes in shopping habits

An EFA was performed to depict shopping styles during the lockdown. Twelve related questionnaire items spanned delivery orders and online shopping. Two items about shopping in person in supermarkets or open markets were unreliable, presumably because open markets were closed in some countries (e.g. Portugal), and supermarkets faced limited capacity and operating hours. As indicated in Table 1, the two extracted components accounted for 60·3 % of the variance (Kaiser–Meyer–Olkin (KMO) = 0·861, *P* < 0·001), and each demonstrated acceptable reliability (i.e. above 0·5) (Brown, 1996).

**Table 1:** Exploratory factor analysis for shopping habits

	Communalities extracted	Variance explained	Mean	sd	Cronbach’s *α*
Delivery orders		41·795			0·886
Orders for pot meals (soup, fish, etc.)	0·511		2·15	1·207	
Orders for fast food (burger, pizza, chips, etc.)	0·770		2·24	1·195	
Delivery price for packed food	0·866		2·70	1·201	
Quantity of orders of packed food for delivery	0·912		2·61	1·197	
Content orders of packaged food for delivery	0·910		2·55	1·159	
Consumption of heavily processed foods	0·711		2·18	1·059	
Consumption of packaged foods	0·506		2·74	1·085	
Online food shopping		18·543			0·554
Online grocery shopping	0·585		2·71	1·352	
Consumption of organic foods	0·746		3·13	1·030	
Consumption of dietary supplements	0·738		2·56	1·211	
KMO = 0·861 Sig.=< 0·001					

KMO, Kaiser–Meyer–Olkin.

Scale: 1, much less; 2, less; 3, neither less nor more; 4, more; 5, much more.

Online shopping and delivery orders differed across countries (Table 2). As shown in Table 3, pairwise tests reinforced these variations: Portugal and China demonstrated a homogeneous shopping behaviour regarding delivery orders. The pairwise tests within Portugal and China do not show significant statistical differences, whereas Turkey and Portugal were homogeneous in online shopping. The frequency of delivery orders remained the same as before the pandemic in Portugal and China but declined in Turkey. In Portugal, online orders started to increase. Nevertheless, the delivery orders became so slow that a supermarket order turned into at least 4 weeks to be delivered.

**Table 2:** Kruskal–Wallis tests

	Kruskal–Wallis test					China		Portugal		Turkey	
	Total *n*	Test statistic	df	Asymptotic sig. (two-tailed test)	Observations	Mean	sd	Mean	sd	Mean	sd
Food preservation											
Dried preservation methods are the same across countries	1065	85·313*	2	0·000	Reject	1·970	0·976	2·481	0·879	2·698	1·155
Bulk preservation methods are the same across countries	1090	43·342*	2	0·000	Reject	3·107	1·008	2·977	0·902	2·649	1·066
Frozen preservation methods are the same across countries	1099	134·149*	2	0·000	Reject	2·525	1·064	2·907	0·911	2·043	0·964
Shopping habits											
Delivery orders are the same across countries	1016	111·476*	2	0·000	Reject	2·675	0·843	2·718	0·779	2·107	0·888
Online food shopping is the same across countries	1033	12·608*	2	0·002	Reject	2·670	0·730	2·812	0·884	2·886	0·954
Food preparation											
Daily time spent cooking is the same across countries	1090	32·481*	2	0·000	Reject	3·135	0·779	3·500	0·649	3·468	0·829
Time spent cooking for future consumption is the same across countries	1020	36·526*	2	0·000	Reject	2·183	0·975	2·281	0·883	2·628	1·081
Cooking logistics are the same across countries	1100	10·033*	2	0·007	Reject	3·285	0·936	3·456	0·695	3·384	0·735
Income variation											
Monthly income variation during the pandemic is the same across countries	1112	19·294*	2	0·000	Reject	2·401	0·898	2·624	0·760	2·628	0·994
Food expenditure											
Variation in total food expenditure during the pandemic is the same across countries	1110	234·598*	2	0·000	Reject	3·395	0·939	2·859	0·525	3·843	1·011
Length of time at home											
The length of time spent at home during the pandemic is the same across countries	1113	118·836*	2	0·000	Reject	4·316	0·807	4·620	0·607	4·809	0·525
Household variation											
The number of persons living in one’s house during the pandemic is the same across countries	1108	4·350*,†	2	0·114	Retain	3·128	0·658	3·034	0·456	3·095	0·605

* The test statistic is adjusted for ties.

† Multiple comparisons are not performed, because the overall test does not show significant differences across samples.

*P* < 0·05; there are statistical differences within the samples.

**Table 3:** Pairwise comparison tests

	Turkey–Portugal			Turkey–China			Portugal–China		
	Test statistic	se	Sig.	Test statistic	se	Sig.	Test statistic	se	Sig.
Food preservation									
Dried preservation methods are the same across countries	49·348	22·158	0·026	199·943	22·057	0·000	150·595	23·752	0·000
Bulk preservation methods are the same across countries	98·392	22·391	0·000	142·340	22·591	0·000	43·948	24·163	0·069
Frozen preservation methods are the same across countries	259·169	22·551	0·000	142·187	22·929	0·000	116·982	24·363	0·000
Shopping habits									
Delivery orders are the same across countries	204·857	22·663	0·000	189·761	21·770	0·000	15·096	24·038	0·530
Online food shopping is the same across countries	16·407	22·676	0·469	75·826	21·866	0·001	59·419	24·211	0·014
Food preparation									
Daily time spent cooking is the same across countries	8·571	22·714	0·706	114·658	23·053	0·000	123·230	24·448	0·000
Time spent cooking for future consumption is the same across countries	90·107	22·550	0·000	123·489	21·433	0·000	33·382	24·034	0·165
Cooking styles									
Cooking styles are the same across countries	185·445	39·470	0·000	4·902	16·743	0·770	190·346	40·032	0·000
Cooking logistics are the same across countries	22·094	22·229	0·320	70·971	22·564	0·002	48·878	24·044	0·042
Income variation									
Monthly income variation during the pandemic is the same across countries	−4·773	21·233	0·822	84·163	21·734	0·000	88·937	23·017	0·000
Food expenditure									
Variation in total food expenditure during the pandemic is the same across countries	340·953	22·273	0·000	137·854	22·779	0·000	203·098	24·130	0·000
Length of time at home									
The length of time spent at home during the pandemic is the same across countries	92·885	18·567	0·000	207·379	19·035	0·000	114·494	20·122	0·000
Household variation									
The number of persons living in one’s house during the pandemic is the same across countries	No differences								

*P* < 0·05; there are statistical differences within the samples.

#### Changes in food preservation

Food preservation also varies culturally. An EFA revealed six items grouped in three components: dried, bulk and frozen. Table 4 shows that all preservation methods differed significantly by country: dried, bulk and frozen. As indicated in Table 3, pairwise comparisons confirmed these variations: only bulk preservation methods were similar in Portugal and China. Drying was most common in Turkey. Bulk methods were slightly higher in China, followed by Portugal and Turkey. Freezing was most common in Portugal, followed by China, and to a lesser extent in Turkey. These results reveal the cultural traditions of those countries and the cooking styles. Bulk preservation may help a healthy life in Portugal and China as it is a traditional method of preserving vegetables in bulk storage by using salt-free techniques. There is no fermentation, either. In Turkey, other than frozen food, there is a culture of drying vegetables and fruits in the summer for consumption in the winter. These methods are more cost-effective than buying fresh foods, regardless of country.

**Table 4:** Exploratory factor analysis for food preservation habits

Factor labels	Factor loading	Mean	Variance explained	*α*
Dried preservation			30·278	0·898
Vegetables dried in the summer for future consumption	0·938	2·50		
Fruits dried in the summer for future consumption	0·938	2·32		
Bulk preservation			29·635	0·877
Fresh fruits in bulk to store for future consumption	0·907	2·93		
Fresh vegetables in bulk to store for future consumption	0·926	2·82		
Frozen preservation			28·925	0·836
Frozen food for daily consumption	0·917	2·30		
Frozen food to store for future consumption	0·907	2·59		
KMO = 0·606 Sig.=< 0·001				

KMO, Kaiser–Meyer–Olkin.

Scale: 1, much less; 2, less; 3, neither less nor more; 4, more; 5, much more.

**Table 5:** Exploratory factor analysis for food preparation

Factor labels	Factor loading	Mean	Variance explained	*α*
Time			34·824	0·835
Time spent cooking breakfast	0·792	3·37		
Time spent cooking lunch	0·782	3·37		
Time spent cooking dinner	0·835	3·64		
Time spent preparing/cooking snacks	0·638	2·96		
Daily time spent preparing/cooking food compared with the previous year	0·779	3·56		
Future consumption			29·857	0·812
Baking home-made bread	0·594	2·67		
Canning food in the summer for future consumption	0·748	2·40		
Cooperating with friends and relatives to prepare food in bulk for future consumption	0·898	2·36		
Helping others prepare food in bulk for future consumption	0·886	2·41		
KMO = 0·826 Sig.=< 0·001				

KMO, Kaiser–Meyer–Olkin.

Scale: 1, much less; 2, less; 3, neither less nor more; 4, more; 5, much more.

#### Changes in food preparation

Food preparation items from the questionnaire were reduced using an EFA, which collapsed the nine items into two groups that collectively accounted for 64·5 % of the variance (KMO = 0·826, *P* < 0·001), KMO tests the consistency of the factorial analysis depicted (Table 5). Food preparation varied significantly across countries regarding daily cooking time and food preparation for future consumption (Table 2). Table 3 lists pairwise tests, showing that Portugal and Turkey were similar in daily cooking time. Portugal and China were similar in time spent on food preparation for future consumption. Daily cooking time increased for all three countries, with Portugal and Turkey registering higher increases than China. Food preparation for future consumption was similar to before the pandemic (Table 2). For instance, Rodrigues *et al.*^(7)^ justify that Portuguese families like to spend time eating with the family. It is also very traditional to spend time preparing meals with the family during holidays or festive seasons. It is not surprising that this habit has been extended now that all the family is at home.

**Table 6:** Exploratory factor analysis for cooking styles

	Communalities extracted	Variance explained	Mean	sd	Cronbach’s *α*
Cooking style		45·003			0·834
Type of food consumed	0·83		2·96	1·04	
Way of cooking	0·88		2·71	1·03	
Type of food cooked	0·89		2·76	1·08	
Cooking logistics		31·839			0·719
Storage space	0·89		3·38	0·96	
Kitchen utensils/equipment at home	0·89		3·34	0·87	
KMO = 0·646 Sig = 0·000					

KMO, Kaiser–Meyer–Olkin.

Scale for cooking style: 1, much more similar; 2, more similar; 3, much more different; 4, neither similar nor different; 5, more different.

Scale for cooking logistics: 1, much less; 2, less; 3, neither less nor more; 4, more; 5, much more.

#### Changes in cooking style

Cooking style items were reduced with an EFA that classified the five items into two groups, accounting for 76 % of the variance (KMO = 0·826, *P* < 0·001) (Table 6). Cooking styles and cooking logistics varied across countries (Table 2). We assume the procedures to prepare the food for cooking styles, whereas cooking logistics refers to the equipment to cook with. These differences were confirmed by pairwise comparison tests aside from Turkey and China, which demonstrated similar cooking styles. Portugal and Turkey had similar cooking logistics. As shown in Table 3, cooking styles were primarily different in Turkey but the same as before the pandemic in Portugal and China. Cooking logistics also varied before and during the pandemic in terms of the need for more space and equipment/utensils.

#### Changes in food expenditure

Food expenditure increased during the pandemic at different paces within countries (*t =* 234·598, *P* < 0·001; Table 2). With an average of 3·84, Turkey demonstrated the most significant increase in food expenditure, followed by China (3·39) and Portugal (2·85). Pairwise comparison tests confirmed that all three countries presented different consumption patterns during the pandemic (Table 3).

#### Changes in length of stay at home

Individuals from these countries spent more time at home during the pandemic but at different rates (Table 2). Pairwise comparisons suggested that all residents spent much more time at home due to lockdowns (Table 3).

### Part II – effects of changes in food habits on healthy eating

COVID-19 has drastically altered the social, economic and psychological spheres of life. It has also affected individuals’ indoor and outdoor activities. Lockdowns have moved some individuals towards healthier habits. Accordingly, this research also aimed to test how such changes influenced healthy eating habits. Explanatory variables included food preparation (time to prepare meals and to cook to consume in the future, cooking styles and cooking logistics), preservation habits (dried, bulk and frozen) and shopping habits (ordering for delivery and shopping online) to explain individuals’ healthy eating, length of time spent at home during the pandemic, household changes, changes in food expenditure, and satisfaction with life and physical activity. The general model accounted for 16·9 % of the variance in healthy eating habits. A likelihood ratio test with 14 df (*n* 119) was 305·78. Ten out of fourteen variables had significant *β* weights. Cooking for future consumption, cooking style, drying as food preservation and household changes were not significant.

The *β* sign and coefficient reflected how variables influenced respondents’ healthy eating habits. Influential items included time to prepare meals, cooking logistics, bulk preservation, online shopping, time spent at home, food expenditure, satisfaction with the ‘new normal’ and physical activity. These results suggested that healthy habits were reinforced through more time cooking, better cooking logistics, bulk preservation, online shopping, more time at home, a higher groceries budget, more physical activity and satisfaction with the ‘new normal’. Items with harmful effects were freezing food and ordering food for delivery, reducing individuals’ healthy food habits.

The model estimated for Turkey accounted for 19·42 % of the variance with a likelihood ratio test with 14 df (*n* 449) of 185·59 (*P <* 0·05). For Portugal, the proportion of variance explained was 42·5 %, and the likelihood ratio for a sample of 315 was 24·30. For China, the variance explained was 18·6 %, and the likelihood ratio was 142·78 for a sample of 319.

As indicated in Table 7, seven variables were significant for Turkey. Healthy food habits arose from more time spent cooking, bulk preservation, online shopping, higher food expenditure, more physical activity and greater satisfaction with the rules of the ‘new normal’. Ordering for home delivery had a negative impact on healthy eating habits. In Portugal, six variables were significant. Items positively affected perceived healthy eating habits were bulk preservation, time spent at home, satisfaction with the ‘new normal’ and physical activity. Items with a negative impact were cooking logistics and freezing food. Seven variables were significant for China. The following elements positively influenced healthy eating habits: cooking style, cooking logistics, bulk preservation, time spent at home, food expenditure and physical activity.

**Table 7:** Results of the ordered probit model

Healthy eating habits	General				China				Portugal				Turkey			
	Coef.	se	*Z*	*P* > *Z*	Coef.	se	*Z*	*P* > *Z*	Coef.	se	*Z*	*P* > *Z*	Coef.	se	*Z*	*P* > *Z*
Food preparation																
Time	0·176	0·074	2·40	0·016	0·146	0·119	1·24	0·217	0·237	0·740	0·32	0·748	0·338	0·106	3·20	0·001
Future	−0·062	0·055	−1·13	0·259	−0·046	0·106	−0·43	0·667	0·188	0·500	0·38	0·706	-0·106	0·071	−1·50	0·135
Cooking style																
Cooking style	0·058	0·047	1·22	0·221	0·159	0·068	2·33	0·020	−0·505	0·488	−1·03	0·302	0·058	0·075	0·77	0·439
Cooking logistics	0·171	0·055	3·13	0·002	0·290	0·105	2·77	0·006	−0·145	0·072	−2·01	0·045	0·081	0·072	1·11	0·266
Preservation habits																
Dried preservation	−0·050	0·049	−1·01	0·313	−0·199	0·093	−2·13	0·034	−0·445	0·420	−1·06	0·289	0·076	0·066	1·15	0·250
Bulk preservation	0·236	0·047	5·05	0·000	0·195	0·076	2·56	0·010	0·266	0·063	4·25	0·000	0·209	0·067	3·12	0·002
Frozen preservation	−0·129	0·049	−2·67	0·008	−0·074	0·068	−1·08	0·280	−0·645	0·082	−7·78	0·000	−0·113	0·078	−1·46	0·145
Shopping habits																
Delivery orders	−0·168	0·061	−2·78	0·005	0·083	0·102	0·82	0·414	−1·099	0·652	−1·69	0·092	−0·344	0·089	−3·84	0·000
Online shopping	0·150	0·061	2·46	0·014	−0·057	0·114	−0·50	0·615	0·1492	0·075	1·98	0·048	0·198	0·078	2·54	0·011
Length of time at home	0·206	0·047	4·38	0·000	0·393	0·099	3·95	0·000	0·293	0·079	3·71	0·000	0·097	0·061	1·58	0·114
Household variation	0·049	0·039	1·26	0·208	0·139	0·084	1·64	0·101	0·294	0·336	0·88	0·379	0·047	0·051	0·93	0·351
Food expenditure	0·291	0·059	4·86	0·000	0·249	0·109	2·26	0·024	0·338	0·712	0·48	0·634	0·309	0·079	3·90	0·000
Happy with life	0·205	0·047	4·37	0·000	0·145	0·082	1·76	0·079	1·017	0·472	2·15	0·031	2 0·27	0·062	3·65	0·000
Physical activity	0·238	0·047	5·06	0·000	0·387	0·089	4·31	0·000	0·373	0·059	6·22	0·000	0·200	0·064	3·14	0·002

*P* < 0·05; the coefficients are statistically significant.

To determine whether healthy eating habits varied across China, Portugal and Turkey, Student’s *t* tests were estimated among the *β* regressors at a 95 % CI (Table 8). Turkey and Portugal demonstrated statistically significant differences in food preparation for future consumption, cooking styles, cooking logistics, drying as preservation and household changes; however, the *β* coefficients of these models were not significant. *β* weights showed opposite signs in both models. Therefore, within Portugal and Turkey, healthy eating habits are quite different. More specifically, the difference in *β* regressors was 11 *v*. 4 between Turkey and China and between Portugal and China.

**Table 8:** Student’s *t* test for *β* coefficients

Healthy eating habits	Turkey–Portugal		Turkey–China		Portugal–China	
	Student’s *t* test	*P* > *Z*	Student’s *t* test	*P* > *Z*	Student’s *t* tesr	*P* > *Z*
Food preparation						
Time	0·447	0·672	0·098	0·539	0·778	0·78
Future	−2·860	0·001	2·576	0·015	10·517	0·000
Cooking style						
Cooking style	5·536	0·000	−3·743	0·000	−3·222	0·000
Cooking logistics	8·850	0·000	−0·779	0·293	−1·116	0·21
Preservation habits						
Dried preservation	6·837	0·000	−0·874	0·270	−0·981	0·25
Bulk preservation	−2·597	0·01	0·132	0·390	0·358	0·37
Frozen preservation	17·632	0·000	−4·682	0·000	−3·289	0·000
Shopping habits						
Delivery orders	4·395	0·000	−1·369	0·16	−2·553	0·02
Online shopping	1·636	0·10	0·706	0·31	9·073	0·000
Length of time at home	−10·245	0·000	−0·099	0·40	−0·136	0·39
Household variation	−5·155	0·000	1·165	0·20	1·305	0·17
Food expenditure	−0,152	0·39	0·085	0·40	0·275	0·38
Satisfaction with rules of the new normal	−8916	0·000	1·795	0·08	1·874	0·07
Physically active	−8658	0·000	−0·011	0·40	−0·019	0·40

*P* < 0·05; there are statistical differences within the samples.

## Discussion and conclusions

The COVID-19 pandemic has altered individuals’ routines as lockdowns have forced people to stay home. Noticeable changes in eating behaviour during the pandemic have involved home cooking and spending on at-home food consumption^(29)^. Using a survey administered in China, Portugal and Turkey, the current study empirically investigated how food purchases, preparation, cooking and expenditure have changed based on the pandemic. Comparing findings across these countries revealed specific insights into each country’s characteristics. In particular, an ordered probit model demonstrated how changes in individuals’ food habits had influenced their healthy eating habits to some extent.

First, people’s shopping habits during the pandemic were explored based on ordering for delivery and shopping online. Findings support research showing that the pandemic has drastically altered people’s shopping habits, leading them to rely on online shopping and delivery^(11,12)^. Portugal and China seemed to have similar shopping behaviour in ordering for delivery. Online shopping slightly increased in all of the three analysed countries. Turkey and Portugal do not present statistically significant differences regarding shopping online. Changes in shopping habits reflect the difference across countries regarding lockdown restrictions and the COVID-19 pandemic in general. Online shopping and ordering for delivery could help individuals spend less time in public. On the other hand, in Turkey, in-person grocery shopping was the only possibility to be outside during the confinement, so people still might prefer to do grocery shopping in person. Moreover, online grocery shopping is available mainly in urban areas. In addition, since the level of food expenditure has increased, people might prefer to visit discount supermarkets not offering online shopping in Turkey.

Second, people prefer different food preservation methods, such as dry, bulk and frozen, based on cultural traditions. For example, China and Portugal similarly favoured bulk preservation, Turkey preferred drying methods and the Portuguese also enjoyed freezing, followed by the Chinese people. During the pandemic, since working from home as possible, many people living in big cities moved to their summer houses, mainly in the coastal parts of Turkey. Also, some of them moved to their rural hometown. Living in smaller towns made it possible to follow some traditional food preservation methods like sun drying. Seasonal and local fruits and vegetables in the coastal or rural areas were easily accessible and abundant, so many urban populations re-discovered traditional ways of living. We did not ask respondents which food they applied preservation methods to. However, traditionally in Turkey, fruits like apples, apricots and plums and vegetables like okra, tomato, eggplant and pepper are sun-dried and stored for winter use. A possible explanation for why the Chinese and Portuguese preferred bulk preservation is that many stayed in their urban areas, and bulk preservation was the feasible food preservation method for them. COVID-19 may have led people to focus on freezing due to spending extra time at home. Moreover, after the declaration of the pandemic, many supermarkets faced stock problems due to panic buying, and thus uncertainty and fear led many people to stockpile^(38)^. To stock up and store fresh foods such as fruits, vegetables, meat, etc., for much more extended periods, people might have started to apply traditional preserving methods like home drying and freezing, because the study results indicated that responders used less frozen food for daily consumption which suggests that they prepared and stored frozen foods for future consumption.

Third, two main factors were identified as relevant to food preparation (i.e. cooking time and future consumption). Significant differences among these countries could offer greater insights into people’s habits^(21,31)^. Portugal and Turkey were similar in cooking time, whereas Portugal and China were similar in preparation for future consumption. As the length of time spent at home increased, it was not surprising that time spent cooking also increased in all three countries, because one of the main barriers to home food preparation/cooking is lack of time^(19)^. Our findings align with previous studies, which reported more time spent cooking in Italy, Denmark, Poland and China^(34,39,40)^. The level of food expenditure also rose during the pandemic, most notably in Turkey, followed by China and Portugal.

Consumers consider home cooking healthy, but the lack of time is the main barrier to home cooking^(19)^. Consistent with previous research, a positive association between more time for cooking and healthy eating habits among Turkish respondents was observed – time spent at home also positively influenced perceived healthy eating among Portuguese and Chinese respondents. Spending more time at home might result in respondents following healthy eating behaviours. Lusk^(36)^ reported that preservation and freshness are indicators of perceived healthiness of food for consumers, and frozen fruits and vegetables were considered less healthy than fresh ones but healthier than canned food.

Our study observed a negative relationship between freezing food and healthy food habits among the Portuguese. This might suggest that the Portuguese preferred to consume fresh foods instead of freezing them for preservation. Bulk preservation of fresh fruits and vegetables is positively associated with healthy eating habits across the three countries in the study. This might suggest that respondents preferred bulk buying fresh fruits and vegetables to visiting grocery shops less often. Many studies reported a decrease in shopping frequency during the pandemic^(3,39)^. Further, fresh fruits and vegetables are defined as healthy by health authorities^(41)^ but also perceived as healthy by consumers^(42)^.

This study integrated the disciplines of tourism and hospitality, marketing, communication, and food science (nutrition) to investigate how COVID-19 has affected people’s food habits across Turkey, Portugal and China. Residents generally shifted to online shopping and delivery services during the pandemic. Insights on food preservation and preparation offer a clearer glimpse into people’s eating habits during the pandemic. Understanding changes in food habits and healthy eating may aid marketers in helping customers adapt to a ‘new normal’ around food-related services (e.g. broader or more intuitive options for online food shopping and delivery; virtual cooking classes to expand one’s culinary repertoire; voluntary training in food preservation methods). This study presents timely empirical evidence to assist policymakers and relevant industry practitioners in coping with events such as pandemics based on individuals’ needs and expectations in different countries. Results also stress the role of national culture in food consumption; associated nuances should be taken into account for policy formulation and practice.

This study has several limitations. First, we assessed habit-based changes in three countries. Although the sample contained a heterogenous group based on respondents’ geographical distribution, the survey was only distributed to people with access to a computer or smartphone. Respondents were also limited to educated residents of urban cities; those in rural areas and below 18 years of age were excluded. Furthermore, the samples by country are not homogeneous, with the Chinese sample comprising more young people than the Portuguese and Turkish samples. As a result, the findings cannot be generalised to other populations in these countries. Future studies should extend this research to a more global level.

Moreover, because changes in healthy eating behaviour constitute a long-term process, longitudinal studies could thoroughly reveal the impacts of the pandemic across individuals’ broader life contexts. Furthermore, in line with Wen *et al.*’s^(43)^ call for interdisciplinary social science research on the pandemic’s impacts on specific industries and populations, more studies should explore food-related topics. One avenue to consider involves the relationship between food and public health – particularly as COVID-19 remains a global health concern.
